# Practical approaches to building up a cardiorenal clinic

**DOI:** 10.1093/ckj/sfac258

**Published:** 2022-12-07

**Authors:** Rafael de la Espriella, Marta Cobo Marcos, Claudio Ronco, Debasish Banerjee, Miguel González, José Luis Górriz, Borja Quiroga, María José Soler, Javier Díez, Julio Núñez

**Affiliations:** Department of Cardiology, Hospital Clínico Universitario de Valencia, Valencia, Spain; Department of Cardiology, Hospital Universitario Puerta de Hierro Majadahonda, Madrid, Spain; Centro de Investigación Biomédica en Red en Enfermedades Cardiovasculares, Madrid, Spain; Department of Medicine, University of Padova, Padova, Italy; International Renal Research Institute of Vicenza, Vicenza, Italy; Department of Nephrology, San Bortolo Hospital, Vicenza, Italy; Renal and Transplantation Unit, St George’s University Hospitals National Health Service Foundation Trust, London, UK; Cardiology Clinical Academic Group, Molecular and Clinical Sciences Research Institute, St George’s, University of London, London, UK; Department of Nephrology, Hospital Clínico Universitario de Valencia, Valencia, Spain; Department of Medicine, Universitat de Valencia, Valencia, Spain; Department of Nephrology, Hospital Clínico Universitario de Valencia, Valencia, Spain; Department of Medicine, Universitat de Valencia, Valencia, Spain; Department of Nephrology, Hospital Universitario de la Princesa, Madrid, Spain; Department of Nephrology, Vall d’Hebron University Hospital, Universitat Autònoma de Barcelona, Nephrology and Kidney Transplant Research Group, Vall d’Hebron Research Institute, Barcelona, Spain; Centro de Investigación Biomédica en Red en Enfermedades Cardiovasculares, Madrid, Spain; Department of Cardiology, University of Navarra Clinic, Pamplona, Spain; Department of Nephrology, University of Navarra Clinic, Pamplona, Spain; Program of Cardiovascular Diseases, Center of Applied Medical Research, University of Navarra, Pamplona, Spain; Department of Cardiology, Hospital Clínico Universitario de Valencia, Valencia, Spain; Centro de Investigación Biomédica en Red en Enfermedades Cardiovasculares, Madrid, Spain; Department of Medicine, Universitat de Valencia, Valencia, Spain

**Keywords:** cardiorenal clinics, cardiorenal disease, cardiorenal program, heart failure, kidney disease

## Abstract

The population with concomitant heart and kidney disease (often termed ‘cardiorenal’ disease) is expected to grow, significantly impacting public health and healthcare utilization. Moreover, the cardiorenal nexus encompasses a bidirectional relationship that worsens prognosis and may complicate pharmacological management in often elderly and frail patients. Therefore, a more cohesive multidisciplinary team approach aiming to provide holistic, coordinated and specialized care would be a positive shift towards improving patient outcomes and optimizing healthcare resources. This article aims to define the organizational aspects and key elements for setting up a multidisciplinary cardiorenal clinical program as a potential healthcare model adapted to the particular characteristics of patients with cardiorenal disease.

## Graphical Abstract

**Graphical Abstract:**
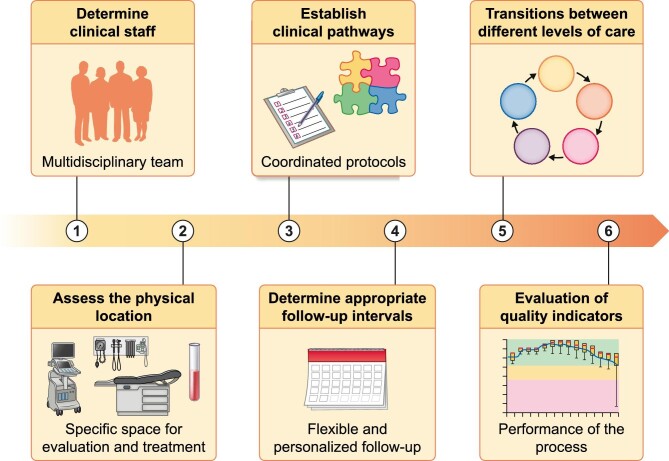

## THE NEED FOR CARDIORENAL PROGRAMS

Heart failure (HF) is a major public health problem associated with high use of resources and healthcare costs [1]. Although the incidence has remained stable or even slightly declined over time, the prevalence is projected to increase due to population longevity and an increase in cardiovascular risk factors and associated comorbidities [2, 3]. Therefore it is a priority to implement solid population healthcare strategies with well-defined objectives throughout the care process. One of these strategies has been the development of specific HF management programs aimed at improving diagnosis, appropriate evidence-based therapy, education and suitable follow-up [4]. However, HF often coexists with relevant comorbidities that worsen prognosis and complicate management, requiring a multidisciplinary team approach to provide holistic, coordinated and specialized care.

Chronic kidney disease (CKD) is one of the most prevalent comorbidities in patients with HF, and at the same time, patients with CKD (especially those with advanced stages) exhibit a high to very high risk for cardiovascular disease (CVD) and incident HF [5–7]. Given the high burden of both conditions and their pathophysiological interrelationship, the cardiorenal nexus represents a real clinical challenge since one condition seems to accelerate the presentation and progression of the other [8–10]. Moreover, despite the undisputed efficacy of current pharmacological treatment options to reduce morbidity and mortality in patients with HF, treatment-induced changes in kidney function are often perceived as deleterious, resulting in ineffective drug implementation. As a result, there is a risk–treatment paradox in managing patients with HF and advanced CKD, such that patients with the highest morbimortality burden are treated with lesser disease-modifying medical therapies [11].

On the other hand, the perception and understanding of kidney disease as a cardiovascular risk factor and as a global cardiovascular risk multiplier has evolved significantly in recent years. However, little progress has been made in developing management structures that offer individualized and coordinated care. Although cardiologists and nephrologists are expected to have advanced knowledge and skills to manage each disease separately, patients are sent back and forth from cardiologists to nephrologists (and vice versa), often leading to conflicting diagnostic and therapeutic approaches. The interaction between cardiologists and nephrologists in the context of an interdisciplinary care model should ensure goal-directed treatment selection (pharmacological and non-pharmacological) based on the individual characteristics of each patient. For instance, an example of that could be the choice of carvedilol/bisoprolol over metoprolol in patients on haemodialysis (HD), the selection of home dialysis therapies, specifically home HD (HHD) or peritoneal dialysis (PD) over conventional HD sessions in patients with concomitant HF or advanced CVD, among many other patient-centred management strategies.

Even though scientific statements support the need for a dedicated cardiorenal multidisciplinary team approach, specific cardiorenal care models are still scarce [12]. Although the barriers to implementing these multidisciplinary care models may vary between countries and healthcare systems, fragmentation of health services, geographic disparities, inadequate infrastructure, insufficient human resources and reluctance to change are some of the most critical and generalized obstacles. In fact, in a recent study conducted in Spain, only 10% of specialized HF clinics reported a specific cardiorenal clinical program and only 30% had established protocols among cardiologists and nephrologists for managing patients with cardiorenal disease [13]. Local, regional and national healthcare providers should support the development of these models of care, ensure the redistribution of resources and facilitate the necessary structural changes to ensure the long-term viability of the cardiorenal clinics. Therefore, efforts should be made to quantify the cost-effectiveness of these models at an institutional level, given that spreading its results may enhance the dissemination of cardiorenal clinic initiatives at a regional level. For instance, Nguyen *et al*. [14] showed that a novel interdisciplinary cardiorenal clinic improved guideline-recommended medication prescription and iron status in 124 patients with HF and advanced CKD. Moreover, Sankaranarayanan *et al*. [15] described the usefulness of a monthly cardio-nephrology meeting to provide expert consensus decision-making, reducing unnecessary outpatient visits. Specialized cardiorenal care has also been developed in other clinical scenarios with promising results, such as in hospitalized patients with concomitant heart and kidney disease or in high-risk kidney transplant candidates [16, 17].

In this article we aim to define the organizational aspects and key elements that a cardiorenal program should have to improve the management of patients with cardiorenal disease.

## DEFINITION AND OBJECTIVES

### Definition

The cardiorenal clinical program is a specialized care model, defined as a set of coordinated and multidisciplinary interventions designed to systematically address the specific management and clinical follow-up of patients with cardiorenal disease.

### Primary objective

The primary goal of cardiorenal clinical programs is to offer a comprehensive and coordinated clinical approach, providing a more efficient and structured model that guarantees personalized and optimized care, reducing clinical variability and offering a faster response capacity, with the ultimate goal of improving patient outcomes and quality of life and, at the same time, reducing healthcare costs.

### Specific objectives

#### Operational considerations

Cardiorenal clinics should be patient-centred, adapting available resources (infrastructure, facilities, staff and finances) and administrative policies to the patient’s needs (Table 1). The following characteristics and components should be considered to build a solid, accessible and functional program (Table 2).

**Table 1:** Specific objectives.

1. Improve communication and coordination between specialists involved in the management of patients with cardiorenal disease at the different health care levels
2. Guarantee continuity of care through collaborative teamwork according to patient needs and disease stage severity
3. Develop common protocols and structured clinical pathways
4. Ensure equity of care
5. Optimize patient flow to enable timely, efficient, and high-quality care
6. Reduce clinical variability, applying current clinical practice criteria and agreed protocols
7. Optimize pharmacological and device-based treatment according to current clinical practice recommendations and individualize therapies according to eGFR strata
8. Ensure the best pharmacological treatment selection/combination based on the individual characteristics of each patient
9. Educate patients and caregivers in self-care
10. Facilitate access to advanced treatment options; supportive and palliative care
11. Reduce the number of patient encounters with healthcare with more time at home
12. Promote telemedicine tools to improve monitoring, enhance communication through the different care levels, and optimize health resources
13. Reduce the number of emergency room visits
14. Promote multidisciplinary research and specific training

**Table 2:** Checklist to consider when building a cardiorenal clinic.

Mandatory
1. Cardiology and nephrology hospitalization wards
2. Availability of kidney replacement therapy
3. Hospital-day setting with a dedicated space for ambulatory parenteral treatment administration (i.e. diuretics, intravenous iron) and managing KRT-derived procedures and complications
4. Readily accessible laboratory monitoring
5. Clinic appointment structure
6. Ultrasound equipment and bioimpedance monitor system
7. Clinical practice protocols
Optional
8. Educational materials and resources for patients and caregivers
9. Virtual visit infrastructure
10. Build or join a research network

#### Clinical staff

Although staffing models may vary according to local healthcare structure, a nephrologist, HF specialist and specialized cardiorenal nurses are critical to the functioning of any cardiorenal clinical program. Supplementary Table 1 summarizes the proposed qualification standards that a cardiorenal nurse should have. Ideally, all members of the cardiorenal team should be present during each clinical visit to provide holistic and coordinated management in order to reduce clinical variability. Furthermore, patients with cardiorenal disease often have multiple comorbidities and geriatric domain impairments that adversely affect their prognosis. Accordingly, coordinating care with other professionals with unique yet complementary expertise is essential to reduce care fragmentation and improve outcomes. Members of such a multidisciplinary and allied care team may include, but are not limited to, a primary care clinician, social worker, dietitian, pharmacist, physical therapist, vascular/dialysis access surgeon, urologist, transplantation team and palliative care clinician.

Another important aspect when organizing interdisciplinary care models is determining who will lead the program. From an operational perspective, and until a dedicated cardionephrologist subspecialty becomes available, cardiorenal clinics should use a model centred around both specialties, with shared leadership in ensuring the delivery of high-quality, safe and evidence-based patient care.

#### Assess the physical location of the cardiorenal clinic

Cardiorenal clinical programs need to be attached to centres with both cardiology and nephrology hospitalization wards, HF outpatient clinics and the availability of kidney replacement therapy (KRT). In addition, considering the relevance of congestion, anaemia and iron deficiency (both absolute and functional) in patients with cardiorenal disease, cardiorenal clinics should have a dedicated space where patients can be comprehensively evaluated using multiparametric tools (i.e. echocardiography or ultrasound equipment, bioimpedance monitoring system), receive intravenous therapy, and have readily accessible laboratory monitoring. For patients included in the PD or HHD programs, educational materials and resources and a dedicated space for training and monitoring should be readily available to patients and their family members.

#### Referral criteria

Although it is unclear which patients are most likely to benefit from being followed in a multidisciplinary cardiorenal clinic, available evidence suggests that patients with stage 4–5 CKD [18, 19] and those with HF with high-risk features may benefit the most [20]. In addition, relatively large observational registries have shown a significant association between incident cardiovascular-related hospitalizations and an accelerated kidney function decline, particularly in patients with lower baseline estimated glomerular filtration rate (eGFR) [21, 22]. Moreover, patients with combined HF and advanced CKD are precisely the most vulnerable to adverse clinical events and in whom disease-modifying therapies have been classically underused because of concerns of kidney-related adverse events [11]. Accordingly, and although further studies are needed to confirm the best entry criteria, we propose targeting the following patient profiles:

1 Inpatient
a Patients with a prior history of very high-risk CKD [eGFR <30 ml/min/1.73 m^2^ or eGFR 30–44 ml/min/1.73 m^2^ and urine albumin:creatinine ratio (UACR) >30 mg/g] or CKD progression discharged from the hospital with uncorrected or persistent cardiovascular conditions (i.e. stage C or D HF, non-revascularized ischaemic heart disease, uncorrected valvular heart disease).
b Patients discharged from the hospital with uncorrected or persistent cardiovascular conditions (i.e. stage C or D HF, non-revascularized ischaemic heart disease, uncorrected valvular heart disease) who developed stage 2 (creatinine ≥2 times baseline or urine volume <0.5 ml/kg for ≥12 h) or stage 3 (creatinine ≥3 times baseline or increase to ≥4.0 mg/dl or acute dialysis, or urine volume <0.3 ml/kg for ≥24 h) acute kidney injury (AKI) according to the current Kidney Disease: Improving Global Outcomes (KDIGO) definition, or stage 2–3 acute kidney disease during the 7- to 90-day period after the initial AKI.
c Patients who required transient aquapheresis procedures due to refractory congestion during admission.
2 Outpatient 
a Patients with a history of uncorrected or persistent cardiovascular conditions (i.e. stage C or D HF, non-revascularized ischaemic heart disease, uncorrected valvular heart disease) with concomitant very high-risk CKD (eGFR <30 ml/min/1.73 m^2^ or eGFR 30–44 ml/min/1.73 m^2^ and UACR >30 mg/g) or CKD progression.
b Patients with very high-risk CKD (eGFR <30 ml/min/1.73 m^2^ or eGFR 30–44 ml/min/1.73 m^2^ and UACR >30 mg/g) or rapidly progressive CKD who develop either acute or progressive high-risk cardiovascular conditions.
c Patients with HF and refractory congestion in whom intensive diuretic treatment is ineffective to achieve euvolemia and who might be considered for KRT or intermittent aquapheresis programs.
d Patients with cardiorenal disease who require a transplant workup (heart, kidney or combined).
e Patients who require consensus decision-making regarding pharmacological or device therapy in ‘gray-zone’ areas (e.g. eGFR <30 ml/min/1.73 m^2^).
f Hyperkalaemia due to renin–angiotensin–aldosterone system (RAAS) inhibitors.

#### Referral sources, follow-up and monitoring

**Figure 1:**
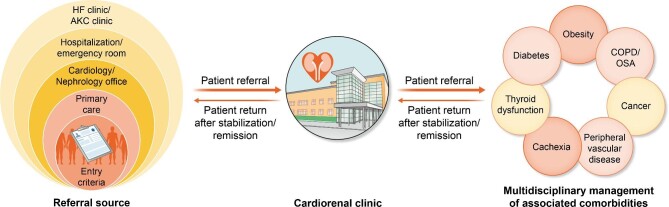
Referral sources and multidisciplinary evaluation and management of related comorbidities.

Considering that patients fulfilling these entry criteria generally correspond to a high-risk population, referrals will probably originate predominantly from HF clinics and advanced kidney care clinics, followed by hospitalization wards, emergency departments, general cardiologists/nephrologists and a small proportion from primary care physicians (Fig. 1). 

Once a patient has been included in the program, it is important to determine appropriate follow-up intervals. For example, stable patients not included in KRT programs and with minimal symptoms should be seen at intervals no longer than 6 months to ensure they are receiving the optimal doses of guideline-directed therapies and are adhering to their medication regimen and to check for symptoms, laboratory monitoring and prevention and treatment of CKD-related complications such as anaemia, acidosis and metabolic disorders. Conversely, shorter follow-up intervals might be necessary for patients recently discharged from the hospital, those with advanced or rapidly progressive CKD or worsening symptoms, patients included in KRT programs or those undergoing up-titration of cardio- or nephroprotective medication. We encourage developing an individualized standard operating procedure to properly define and formalize the components and coordinated processes that the cardiorenal team needs to perform (Table 3). Although onsite visits are the preferred review modality for most patients, different ways of communication (e-consult, telemedicine) should also be offered to patients and caregivers to monitor laboratory results, assess treatment adherence, evaluate drug-related adverse effects and facilitate early detection of decompensations and management of possible adverse outcomes.

**Table 3:** General considerations to be included in standard operating procedures.

**Referral criteria**	
**Inpatient**	**Outpatient**
1. Patients with CKD stages ≤G3bA2 or CKD progression prior to admission who are discharged with uncorrected or persistent cardiovascular conditions 2. Patients discharged with uncorrected or persistent cardiovascular conditions who developed persistent stage 2–3 AKI according to the KDIGO definition or stage 2–3 AKD during the 7- to 90-day period after the initial AKI 3. Patients who required transient aquapheresis procedures due to refractory congestion during the index admission	1. Patients with prior history of uncorrected or persistent cardiovascular conditions with concomitant CKD stages ≤G3bA2 or CKD progression 2. Patients with CKD stages ≤G3bA2 or CKD progression who develop either acute or progressive high-risk cardiovascular conditions 3. Patients with refractory congestion who might be considered for KRT or intermittent aquapheresis programs 4. Patients with cardiorenal disease who require transplant workup (heart, kidney, or combined) 5. Patients who require consensus decision-making regarding pharmacological or device therapy in ‘gray-zone’ areas (e.g. eGFR <30 ml/min/1.73 m^2^)
**Follow-up and monitoring**	
1. Early follow-up visit (7–30 days) • Patients recently discharged from the hospital (ideally within the first 7–10 days post-discharge) • AKI/AKD • Worsening symptoms/persistent congestion • Cardio- or nephroprotective treatment initiation/up-titration 2. Short follow-up intervals (1–3 months) • Patients included in KRT programs or transplant workup • Rapidly progressive CKD or CKD stages G4–G5 • More than two worsening HF events within the last year despite optimal medial and device therapy • Patients refractory/intolerant to GDMT 3. Long follow-up intervals (6 months) • Stable eGFR (if eGFR >30 ml/min/1.73 m^2^) and UACR • Improving heart/kidney function and biomarker profile • Stable symptoms/signs • Optimal GDMT	Systematic checklist evaluation to be performed in each follow-up clinical visit Multiparametric assessment of congestion Kidney function, electrolyte, and acid-base monitoring Assess drug-related adverse events Medication reconciliation Initiation or up-titration of GDMT Monitoring and treatment of CKD-related complications (i.e. anaemia, iron deficiency, mineral and bone disorder) Reinforce patient and caregiver education Risk stratification and care planning Provide non-pharmacological advice Identify the need for, coordinate and provide palliative care
Flexibility to modify the frequency of follow-up as needed based on the patient's trajectory, needs and stage of the disease	

AKD: acute kidney disease; GDMT: guideline-directed medical therapy.

In addition, considering the high prevalence of associated comorbidities on a population level, we propose developing well-designed clinical care pathways that guarantee a dynamic flow of patients between the cardiorenal clinic program, primary care and other clinical specialties within and outside of cardiorenal medicine to ensure adequate attention to comorbid conditions and non-cardiovascular preventive care (Fig. 1).

#### Transitions between different levels of care

**Figure 2:**
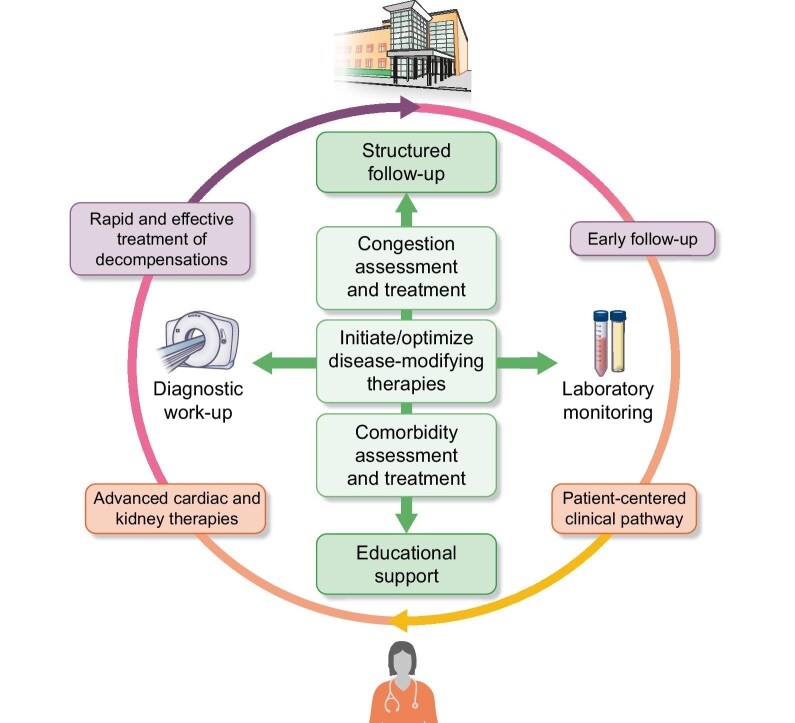
General follow-up approach.

One of the most important aspects of any multidisciplinary clinical model that offers integrated care is the subsequent follow-up and transition from different levels of care depending on the disease stage and progression [23]. Therefore it is imperative to develop coordinated and consensed protocols between healthcare providers to improve transition efficiency from one setting to the next (Fig. 2).

##### Hospital–home transition

Early follow-up after discharge is an essential step in the success of the transition from the acute phase to long-term outpatient management [24]. An early follow-up visit should be scheduled, ideally within the first 7–10 days post-discharge. This intervention should include a systematic evaluation using checklists (Table 3) to confirm that the patient is euvolemic; reinforce patient and caregiver support and education; evaluate potential clinical, haemodynamic, renal or electrolyte deterioration; detect drug-related adverse events; reconcile medications and optimize treatment based on current guidelines.

##### Transitions of care in patients with acute exacerbations

Cardiorenal disease encompasses changes in the clinical risk of hospitalization and death over time, with risk increasing from pre- to new-onset cardiorenal syndrome, and further increasing with each episode of decompensation, where there is often a deterioration of kidney function and cardiovascular signs/symptoms requiring hospitalization or outpatient interventions. Therefore cardiorenal clinics should include readily accessible outpatient care (day-hospital setting) to deal with such problems in a timely manner, particularly in the period of close proximity to the worsening event. Importantly, this phase should be based on active communication with other levels of care to identify red flags or implement alert systems that allow cardiorenal team members to be aware of such decompensations and adopt prompt interventions.

##### Transitions of care after clinical/functional stabilization/remission

Although we caution against ‘stable’ cardiorenal disease terminology, some patients exhibit clinical improvement along with resolution/remission of previously present structural and functional heart and kidney disease. In this phase, patients can be discharged from the program as long as the connectivity among existing care networks and transitional care interventions and protocols focus on optimizing disease-modifying therapies are guaranteed. Therefore the cardiorenal clinic should ensure a close relationship with the various participants from different disciplines involved in patient care and determine the relational structures needed to achieve optimal care coordination.

##### Transition towards advanced cardiac and kidney therapies

###### Kidney replacement therapy/kidney transplantation

By monitoring the patient closely through frequent laboratory and clinical evaluations (fluid retention and patient-reported symptoms) when the GFR is decreasing or in cases of persistent and refractory congestion, it is possible to tailor KRT initiation to when both the patient and cardiorenal team feel the timing is optimal. At this point, the cardiorenal team should move away from the traditional ‘one-size-fits-all’ approach and provide more individualized or personalized care offering the best KRT modality according to the patient profile.

###### PD

PD is an excellent mode of KRT for patients with advanced cardiorenal disease, as it provides greater preservation of residual kidney function, continuous ultrafiltration with better haemodynamic tolerability and efficient volume control while concurrently correcting the metabolic consequences of diminished kidney function [25]. Although the optimal timing for PD initiation in patients with cardiorenal disease is unclear, it should be considered when one or more of the following are present: uraemic-related symptoms, inability to control volume status with diuretics or congestion-related organ damage (i.e. congestive nephropathy/hepatopathy in the context of right-sided HF) but well before critical (medically resistant acid–base or electrolyte abnormalities, low residual diuresis, kidney function loss) or long-lasting side effects (wasting and sarcopenia) develop [26, 27]. In this scenario, an incremental PD strategy increasing dialysis dose in a step-wise manner may help preserve residual kidney function while still achieving individualized clearance goals [28]. In addition, the time window between catheter placement and treatment initiation can be used to pre-train the patient and caregivers for the upcoming procedures, reduce intra-abdominal pressure through ascites drainage in those with recurrent ascites and provide a home visit by PD staff to check for hygiene problems and storage capacity for PD materials and solutions. However, although PD is an excellent KRT modality for many cardiorenal patients, clinical complications such as catheter malfunctions, peritonitis and ultrafiltration failure may occur, requiring conversion from PD to HD.

###### Chronic HD

Conventional thrice-weekly in-centre HD is usually not the first-choice KRT modality for most cardiorenal patients because of haemodynamic stability concerns during sessions (which often limits fluid removal) and the possibility of interdialytic volume overload [29]. However, conventional HD is sometimes the only available KRT modality (i.e. urgent requirement for KRT, elderly and frail patients with barriers to self-care, anatomic contraindication to PD, healthcare structure, financing and delivery), and the cardiorenal team may provide supportive care to achieve patient-centred goals. For instance, an integrative and comprehensive assessment of the cardiac function and volume status combining ultrasound techniques, bioimpedance tools and biomarkers (dry weight revisited) may guide the need and the rate of fluid removal, enhancing haemodynamic tolerance [30–32]. Furthermore, HHD is emerging as an attractive modality that may overcome the adverse consequences of interdialytic cycles of hypervolemia coupled with rapid and aggressive ultrafiltration commonly seen with conventional HD [29]. By increasing the frequency (daily) and duration of HD sessions, HHD may decrease the ultrafiltration rate (better haemodynamic tolerance) while ensuring daily fluid and uraemic solute removal [29]. However, both HHD and conventional HD require permanent vascular access [i.e. tunnelled central venous catheter, arteriovenous fistula (AVF) or arteriovenous graft], which confers an additional source of adverse events [33, 34]. Although a mature AVF is generally well tolerated and has the best profile in terms of access-related complications and patency rates, it may have deleterious haemodynamic effects in patients with concomitant heart disease, mainly driven by decreased systemic vascular resistance and increased venous return [35]. This aspect is especially relevant in patients with concomitant right-sided HF and pulmonary hypertension, in which an AVF may accelerate disease progression [36, 37]. In this particular setting, the cardiorenal team should be aware of this negative association and balance the pros and cons of AVF closure in selected cases. Moreover, patients with advanced cardiorenal disease are often not considered eligible for an AVF because of their high burden of comorbid conditions that increase surgical risks, shortened life expectancy and/or poor vasculature. As a result, tunnelled HD catheters are the most commonly used vascular access in this population, increasing the risk of bacteriemia and having the highest rate of vascular access dysfunction [34]. 

On the other hand, emerging evidence supports the feasibility and efficacy of intermittent aquapheresis programs in selected patients for volume control [38].

###### Mechanical circulatory support/heart transplantation

In patients with advanced HF referred for long-term mechanical circulatory support (LT-MCS) or heart transplantation, kidney disease is highly prevalent and is one of the most powerful predictors of post-intervention survival [39]. In fact, advanced kidney dysfunction (eGFR <30 ml/min/1.73 m^2^) deemed ‘irreversible’ and chronic KRT are contraindications for both LT-MCS and heart transplantation [4]. Although distinguishing irreversible forms of kidney dysfunction from likely reversible forms is challenging, a comprehensive and multiparametric evaluation of congestion/hypoperfusion, including point-of-care ultrasound, invasive assessment (i.e. right heart catheterization, invasive blood volume analysers), bioimpedance tools and biomarkers, may provide relevant information regarding which patients are likely to benefit from those therapies [32]. In this scenario, cardiorenal clinics may offer a window for patient optimization before patient eligibility.

##### Transition toward end-of-life care

Patients with advanced cardiorenal disease, particularly those with stage D HF and kidney failure, often have a high symptom burden that substantially affects their health-related quality of life. Furthermore, many of these patients will not be candidates for advanced solutions such as KRT, kidney/cardiac transplantation or mechanical circulatory support. Therefore, at this level of care, the objective is to enhance symptoms and quality of life through a multidisciplinary strategy that includes collaborative decision-making, palliative care planning and psychological and social support. If patients require hospitalization at the end of life because of poor symptom control and/or suboptimal family support, direct admission to a palliative care unit is desirable. In addition, for those included in KRT programs, treatment decisions such as reducing dialysis dose and frequency to a minimum (palliative dialysis) or withdrawing completely from KRT should be individualized according to the patient’s free choice. This whole process should be well defined in the cardiorenal care pathway.

### Specific approaches when managing patients with cardiorenal disease

The suggested specific approach for managing patients with cardiorenal disease is summarized in Table 4.

**Table 4:** Specific approach for managing patients with cardiorenal disease.

Therapy	Recommendation/comment
Congestion	Congestion assessment: multiparametric approach • Identify the predominant phenotype: compartmental distribution (intravascular/tissue)—regional distribution (pulmonary/systemic) • Integrate clinical signs/symptoms, biomarkers (CA125, NT-proBNP), lung ultrasound, VExUS, bioimpedance Acute heart failure with volume overload (inpatient) • Loop diuretics as the first choice: 2–2.5 times oral dose or 60–80 mg as IV bolus in diuretic naïve; subsequent dose according to urinary sodium • Consider 500 mg IV bolus of acetazolamide during the first 3 days of admission to improve decongestion • Consider hypertonic saline solutions • Consider adding an SGLT2I to enhance decongestion and improve outcomes Ambulatory worsening symptoms with volume overload • IV or subcutaneous infusions Oral diuretic therapy optimization: • Loop diuretics as the first choice • Sequential nephron blockade • Consider adding an SGLT2I to enhance decongestion and improve outcomes Changes in creatinine/eGFR should always be seen in the clinical context/status. An increase in creatinine should not stop further decongestive therapy, especially if congestion persists. Caution if doubling serum creatinine. Consider PD or intermittent aquapheresis sessions for selected patients with refractory congestion.
Pharmacological treatment—HF	After initiating RAAS inhibitors, ARNIs or SGLT2Is, a transient decrease in eGFR is expected and should not prompt their interruption • An increase in serum creatinine of <50% above baseline, as long as it is <266 μmol/l (3 mg/dl), or a decrease in eGFR of <10% from baseline, as long as eGFR is >25 ml/min/1.73 m^2^, can be considered as acceptable Specific therapies • ARNI as the first choice in HFrEF, and consider its use in HFmEF • Carvedilol/bisoprolol over metoprolol in patients on KRT • SGLT2I in HF, CKD (eGFR ≥25 ml/min/1.73 m^2^) and type 2 diabetes mellitus • Espironolactone/eplerenone in HFrEF. Consider its use in HFmEF. Caution in patients with eGFR <30 ml/min/1.73 m^2^ or serum potassium >5.0 mmol/l • Vericiguat in patients who had a worsening HF event despite optimal medical therapy. Do not initiate in patients with eGFR <15 ml/min/1.73 m^2^
Comorbidities and related conditions	Atrial fibrillation • Direct oral anticoagulants may be used after appropriate dose adjustment in patients with advanced CKD (eGFR 15–30 ml/min/1.73 m^2^); dabigatran contraindicated if eGFR <30 ml/min/1.73 m^2^ Type 2 diabetes mellitus • GLP1a and SGLT2I as the first choice in patients with type 2 diabetes mellitus • Finerenone in patients with type 2 diabetes mellitus and CKD (eGFR ≥25 ml/min/1.73 m^2^) Hyperkalaemia • Consider new potassium binders (patiromer/sodium zirconium cyclosilicate) CKD-MBD • Monitoring serum calcium and phosphate every 3–6 months (in CKD G3b–G4) and PTH every 6–12 months. Consider shorter intervals in CKD G5 • Avoid hypercalcemia and hyperphosphatemia in CKD G3a–G5 • It is reasonable to reserve the use of calcitriol and vitamin D analogues for patients with CKD G4–G5 with severe and progressive hyperparathyroidism Dyslipidaemia • LDL-C <1.8 mmol/l (70 mg/dl) in high-risk patients and <1.4 mmol/l (55 mg/dl) in very high-risk patients • Avoid rosuvastatin and fibrates in advanced CKD Anaemia • Monitoring the iron status and evaluating the need for IV iron replacement therapy and erythropoiesis-stimulating agents [55] Contrast-induced AKI • Limit large amounts of contrast media • Use either iso-osmolar or low-osmolar iodinated contrast media • Prophylactic hydration (isotonic crystalloids, 1.0–1.5 ml/kg/h) in patients at risk for AKI at least 6 h before and after contrast-enhanced imaging studies or interventions
Advanced cardiac and kidney therapies	Heart transplantation • Assess the potential impact of immunosuppressive therapy on CKD progression Kidney transplantation • The severity of cardiovascular-related conditions is a significant contributor to worse patient and allograft outcomes Mechanical circulatory support
	• Consider the risk:benefit ratio in patients with advanced CKD Implantable cardioverter defibrillator Cardiac resynchronization therapy • Reverse remodelling following CRT is observed across all stages of CKD, yet the response is less pronounced in advanced CKD • HD • Assess the impact on the cardiovascular system/haemodynamics • Consider cardioprotective HD (blood volume control, long sessions) PD • Preferred KRT modality in patients with heart failure and advanced structural heart disease • Consider its use in patients with refractory congestion regardless of eGFR
Palliative care	Primary palliative care • Control pain, dyspnoea and other symptoms • Assess and reduce emotional distress to patient and caregiver • Manage ‘trigger events’ • Predict and communicate prognosis • Choose therapy Specialist palliative care • Consider hospice utilization for advanced patients and end-of-life transition • Consider reducing dialysis dose and frequency (palliative dialysis) or withdrawing completely for KRT

ARNI: angiotensin receptor–neprilysin inhibitor; CA125: antigen carbohydrate 125; CRT: cardiac resynchronization therapy; GLP1a: glucagon-like peptide-1 agonists; HFrEF: heart failure with reduced ejection fraction; HFmEF: heart failure with mildly reduced ejection fraction; LDL-C: low-density lipoprotein cholesterol; NT-proBNP: N-terminal pro-hormone B-type natriuretic peptide; PTH: parathyroid hormone; SGLT2I: sodium–glucose cotransporter-2 inhibitor; VExUS: venous excess ultrasound.

#### Congestion

Fluid overload plays a major role in the pathogenesis, presentation and prognosis of most patients with combined heart and kidney disease and represents a core target for treatment. However, the optimal method to assess fluid status and to determine euvolemia (‘dry weight’) in decompensated HF or kidney disease remains an unresolved issue, resulting in diagnostic uncertainty and hampering therapeutic decision-making. Therefore, one of the most important qualification standards for cardiorenal specialists is a deep understanding of cardiorenal physiology (with a particular focus on intrarenal haemodynamics and the complex and dynamic interplay between the interstitial and intravascular fluid compartments) as well as diuretic pharmacokinetics and pharmacodynamics. Accordingly, one standard operating procedure in each clinical visit should be a comprehensive and multiparametric evaluation of volume status to detect subclinical congestion, better phenotype congestion profiles [32] and offer personalized management [40–43] strategies.

#### Guideline-directed medical therapy implementation in patients with HF

Several pharmacological treatment options, such as RAAS inhibitors, angiotensin receptor–neprilysin inhibitors, mineralocorticoid receptor antagonists and sodium–glucose cotransporter 2 inhibitors, have been shown to reduce morbidity and mortality in patients with HF and reduced ejection fraction and are recommended as a class I indication in clinical practice guidelines [4]. However, as these drugs may induce an initial eGFR decrease [11], clinicians often struggle to initiate or up-titrate these therapies, as any deterioration in kidney function is often perceived as deleterious. In fact, the presence of kidney disease is one of the main reasons for ineffective drug implementation in HF patients [44, 45]. Therefore cardiorenal clinics should offer a structured and personalized follow-up that favours the implementation of these life-saving therapies and provide rapid and efficient solutions to drug-related adverse effects.

#### Anticoagulation in atrial fibrillation (AF)

CKD is associated with a higher prevalence of AF, thromboembolic events and bleeding complications [46]. Anticoagulation has been shown to reduce the risk of stroke and mortality in patients with AF and mild and moderate CKD [47]. However, patients with advanced CKD or those on KRT have been excluded from randomized controlled trials. Observational studies have shown conflicting results, even suggesting that treatment with vitamin K antagonists (VKAs) may be harmful in this end-stage CKD [47]. Other specific problems related to the use of VKAs that should be considered are the increased risk of developing vascular calcification [48] and anticoagulant-associated nephropathy [49]. The available data suggest that direct oral anticoagulants have a better safety and efficacy profile in patients with CKD, but data in patients with advanced CKD are scarce [50]. On the other hand, left atrial appendage occlusion has been shown to reduce the incidence of thromboembolic events in patients with AF, with a low incidence of adverse events, so this strategy should be considered for patients with an increased risk of bleeding [51].

#### Prevention of contrast-induced AKI

Diagnostic and therapeutic procedures that require contrast agents are frequently needed in patients with CVDs. Although pre-existing CKD is the strongest patient-related risk factor for developing contrast-induced AKI [52], the use of high contrast volumes (>350 ml or >4 ml/kg) or repeated exposure to contrast agents (within 72 h) has also been shown to increase the risk [53]. Accordingly, the cardiorenal team should increase the awareness among clinicians to limit large amounts of contrast media, using either iso-osmolar or low-osmolar iodinated contrast media rather than high-osmolar iodinated contrast media, and ensure prophylactic hydration in patients at risk for AKI at least 6 h before and after contrast-enhanced imaging studies or interventions [54].

## PROPOSED CARDIORENAL CARE QUALITY INDICATORS

One of the most important aspects of specific clinical management programs lies in the continuous monitoring of measures designed to evaluate the performance of the process. These indicators should be directed at specific clinical outcomes (i.e. mortality and readmissions) and process outcomes (Table 5).

**Table 5:** Outcome and process indicators.

Outcome indicators	Process indicators
• 0-day, 90-day and 1-year mortality rates (all-cause, cardiovascular, kidney-related) • 30-day, 90-day and 1-year hospital readmission rates (all-cause, cardiovascular, HF-related, kidney-related) • Emergency department visits due to hyperkalaemia, worsening kidney function, HF-related or cardiovascular-related events • Unscheduled visits due to hyperkalaemia, worsening kidney function, HF-related or cardiovascular-related events • Major renal adverse events defined as [56]: – Worsening of kidney disease (sustained >40% reduction in eGFR) at 1 year [26] – Progression to end-stage kidney disease (eGFR <15 ml/min/1.73 m^2^ or sustained initiation of renal replacement therapy) – Renal death (death due to end-stage CKD when renal replacement therapy was not initiated or was discontinued) at 30 days, 90 days and 1 year • Heart or kidney transplantation rates • Percentage of patients who required advanced circulatory support	• Time from external referral to cardiorenal visit • Proportion of patients receiving guideline-based therapies • Proportion of CKD grade 4–5 patients with haemoglobin of 10–12 g/dl [28] • Proportion of patients with adequate iron status [4, 28] • Proportion of patients with hyperphosphatemia • Rate of HD-related complications • Proportion of patients with accurate control of mineral and bone disorder [57] • Proportion of patients with hyperkalaemia • Number of patients initiating KRT • Rate of PD-related complications • Patient satisfaction • Staff satisfaction

## THE NEED FOR SPECIFIC CARDIO-NEPHROLOGY EDUCATIONAL PROGRAMS

Interest in cardiorenal pathology has evolved considerably in recent years. Clinical investigations and research concerning cardiorenal disease have increased significantly, to the point where the constant advance in cardiovascular and renal diseases has positioned cardiorenal medicine as a new discipline [58]. 

However, current nephrology and cardiology training seems insufficient to encompass the complexity of cardiorenal disease and the rapid advances in the field of cardiovascular medicine. Therefore a change in the approach is required. There is a clear need for specific training programs that improve nephrologists’ and cardiologists’ knowledge and skills in all the aspects related to managing patients with cardiorenal disease. Education is also required to promote the culture of shared care and avoid the prejudice of ‘us and them’ that often leads to antagonistic and harmful interventions.

Along this line, leaders in the field have proposed specific road maps to achieve these goals. Structured training programs should be included during the time of specialization in nephrology. However, postgraduate programs and fellowships should also be developed to make the physician fully capable of providing more effective clinical care to this growing and increasingly complex population [59–61]. Moreover, a consensed and comprehensive core curriculum must define the knowledge, skills and competencies to be achieved and cover the specific topics related to cardiorenal disease that should be included in educational resources and assessment tools [59].

In conclusion, large institutions and national/international societies must focus on developing educational resources and promoting shared and structured programs resulting in board certification in cardiology and nephrology. Furthermore, implementing this discipline should be encouraged, favouring optimal care for patients with cardiorenal disease, a closer and more effective collaboration between specialists and the stimulation of specific research that expands knowledge of cardiorenal disease.

## CONCLUSION

In summary, given the complexity and the increasing prevalence of cardiorenal disease, there is a need to develop specialized models of care for patients with combined heart and kidney disease. A multidisciplinary, coordinated and structured approach across the different levels of care may improve patient outcomes and the utilization of healthcare infrastructures and resources. This article provides some organizational aspects and the key elements for setting up a multidisciplinary cardiorenal clinical program as a potential healthcare model adapted to the particular characteristics of patients with cardiorenal disease.

## Supplementary Material

sfac258_Supplemental_File

## Data Availability

All data are incorporated into the article and its online supplementary material.
