# Supplementary material for: Practical approaches to building up a cardiorenal clinic
Source: Clin Kidney J. 2022 Dec 7;16(5):780–92. doi: 10.1093/ckj/sfac258 (PMC10157785; doi:10.1093/ckj/sfac258)
Supplement: sfac258_Supplemental_File [file sfac258_supplemental_file.docx]

Supplementary Table 1. Specific cardiorenal nurse curriculum

| **Specific knowledge and skills** |
| --- |
| 1. Awareness of triggers for clinical or functional deterioration |
| 1. Assess and monitor common symptoms and signs |
| 1. Implement and evaluate an effective patient and caregiver education and identify barriers to patient learning |
| 1. Provide self-care, lifestyle, and non-pharmacological advice |
| 1. Accurately interpret and manage monitoring data and escalate appropriately to a more senior member of the cardiorenal team |
| 1. Knowledge of common drug indications, contraindications, and potential side effects. |
| 1. Effectively discuss with the patient their medication, the action plan for optimizing dose, side-effects of medication, and important interactions with other medication or foods |
| 1. Competently and rapidly identify decompensations and deliver care (i.e., ambulatory parenteral diuretics, intravenous iron, ) |
| 1. Undertake a comprehensive assessment of comorbidities such as frailty, mental functioning, and emotional state using objective measurement tools |
| 1. Knowledge of common peritoneal dialysis procedures, including tests to assess peritoneal membrane function (such as the peritoneal equilibration test) and tests of adequacy |
| 1. Training patients and caregivers to perform continuous ambulatory peritoneal dialysis and provide safety protocols and instructions for the proper usage of the dialysis machines in those who require other forms of ambulatory dialysis (i.e., automated peritoneal dialysis, home hemodialysis) |
| 1. Detect major complications of peritoneal dialysis/ home hemodialysis and take the necessary nursing actions and escalate appropriately to a more senior member of the cardiorenal team |
| 1. Identify the need for, coordinate, and provide care at the end of life to the patient and their family |
| 1. Leadership in cardiorenal nursing |
